# Identifying immune cells-related phenotype to predict immunotherapy and clinical outcome in gastric cancer

**DOI:** 10.3389/fimmu.2022.980986

**Published:** 2022-08-11

**Authors:** Sutian Jiang, Xuzhong Ding, Qianqian Wu, Tong Cheng, Manyu Xu, Jianfei Huang

**Affiliations:** ^1^ Department of Clinical Biobank & The Institute of Oncology, Affiliated Hospital of Nantong University, Nantong, China; ^2^ Department of Pathology, Lishui People’s Hospital, Lishui, China; ^3^ Department of Gastrointestinal Surgery, Lishui People’s Hospital, Lishui, China

**Keywords:** Gastric cancer, Immunotherapy, Prognosis, mIHC, TMA

## Abstract

**Background:**

The tumor microenvironment is mainly composed of tumor-infiltrating immune cells (TIICs), fibroblast, extracellular matrix, and secreted factors. TIICs are often associated with sensitivity to immunotherapy and the prognosis of multiple cancers, yet the predictive role of individual cells on tumor prognosis is limited.

**Methods:**

Based on single-sample gene set enrichment analysis, we combined three Gene Expression Omnibus (GEO) cohorts to build a TIIC model for risk stratification and prognosis prediction. The performance of the TIIC model was validated using our clinical cohort and the TCGA cohort. To assess the predictive power of the TIIC model for immunotherapy, we plotted the receiver operating characteristic curve with the IMvigor210 and GSE135222 cohorts.

**Results:**

Chemokines, tumor-infiltrating immune cells, and immunomodulators differed between the two TIIC groups. The TIIC model was vital for predicting the outcome of immunotherapy. In our clinical samples, we verified that the expression levels of PD-1 and PD-L1 were higher in the low TIIC score group than in the high TIIC score group, both in the tumor and stroma.

**Conclusions:**

Collectively, the TIIC model could provide a novel idea for immune cell targeting strategies in gastric cancer and predict the survival outcome of patients.

## Introduction

Gastric cancer (GC) has a high incidence globally and is one of the leading causes of cancer-related death (1, 2). The 5-year survival rate of patients with gastric cancer is less than 30% (3). The tumor immune microenvironment (TME) has been the focus of cancer research because it brings a series of challenges to accurate diagnosis and individualized immunotherapy (4). Immunotherapy can activate the host’s natural defense system to identify and remove tumor cells and has shown unparalleled survival benefits in various cancers (5). The clinical success of immune checkpoint inhibitors has wholly changed traditional treatment methods. By targeting immune regulatory factors, including the PD-1/PD-L1 axis, the function of effector T cells is enhanced to promote the cytotoxic killing of cancer cells (6). However, most patients do not respond to immunotherapy because of primary or acquired drug resistance (7). A comprehensive and profound understanding of the immune microenvironment may provide more advanced prognostic biomarkers for clinical treatment and reveal novel methods for immunotherapy in GC patients.

Tumor-infiltrating immune cells are highly related to overall survival and treatment response (8). Extensive studies have shown that tumor-infiltrating immune cells play a crucial role in tumor progression, metastasis, and recurrence (8–10). However, the current technologies can only evaluate a small number of tumor-infiltrating lymphocytes. The accuracy of using a single tumor-infiltrating lymphocyte to predict prognosis and treatment response is limited. Thus, we used computational methods to quantify multiple tumor-infiltrating lymphocytes to fully understand the impact of lymphocytes combination on gastric cancer prognosis and immunotherapy response (11). In our research, based on the immune infiltration levels of 24 immune cells combined with the least absolute shrinkage operator (LASSO) and stepwise regression analysis, we constructed a TIIC model for GC patients from the GEO cohort. We also verified the prognostic value of the TIIC model in a few public cohorts, the TCGA cohort, and our clinical cohort. Furthermore, the TIIC model could predict which patients respond to immune checkpoint inhibitors in the IMvigor210 and GSE135222 cohorts.

## Methods

### Public databases

There were seven public cohorts included in our research, including the GSE62254, GSE57303, GSE15459, GSE84437, TCGA, GSE135222, and IMvigor210 cohorts (**Table 1**). GEO datasets were downloaded from Genomic Data Commons (https://www.ncbi.nlm.nih.gov/geo/). We downloaded the IMvigor210 dataset from freely available, fully documented software under the Creative Commons 3.0 license (http://research-pub.gene.com/IMvigor210CoreBiologies). The TCGA mRNA expression matrix, clinical information, and mutation profiling datasets were downloaded from the UCSC Xena browser (https://xenabrowser.net/datapages/).

**Table 1:** Patient demographics.

Characteristics	GEO 562 cohort (GSE62254, GSE57303, GSE15459) (%)	GSE84437 cohort (%)	TCGA cohort (%)	IMvigor210 cohort (%)	GSE135222 cohort (%)	Clinical cohort (%)
No of patients	562 (100)	433 (100)	378 (100)	298 (100)	27 (100)	303 (100)
Age						
<=60	204 (36.43)	194 (44.80)	127 (33.60)	NA	16 (59.26)	111 (36.63)
>60	356 (63.57)	239 (55.20)	251 (66.40)	NA	11 (40.74)	192 (63.37)
Unknown	2					
Gender						
male	377 (67.08)	296 (68.36)	237 (62.70)	NA	22 (81.18)	80 (26.40)
female	185 (32.92)	137 (31.64)	141 (37.30)	NA	5 (18.82)	223 (73.60)
Stage						
I	64 (11.39)	NA	48 (12.70)	108 (36.24)	NA	NA
II;	136 (24.20)	NA	120 (31.75)	75 (25.17)	NA	NA
III	208 (37.01)	NA	169 (44.71)	60 (20.13)	NA	NA
IV	154 (27.40)	NA	41 (10.85)	56 (18.79)	NA	NA
T stage						
T1	NA	11 (2.54)	17 (4.50)	NA	NA	42 (13.86)
T2	NA	38 (8.78)	76 (20.11)	NA	NA	56 (18.48)
T3	NA	92 (21.25)	180 (47.62)	NA	NA	179 (59.08)
T4	NA	292 (67.44)	105 (27.78)	NA	NA	24 (7.92)
N stage						
N0	NA	80 (18.48)	120 (31.75)	NA	NA	118 (38.94)
N1	NA	188 (43.42)	100 (26.46)	NA	NA	48 (15.84)
N2	NA	132 (30.48)	79 (20.90)	NA	NA	68 (22.44)
N3	NA	33 (7.62)	79 (20.90)	NA	NA	69 (22.77)
M stage						
M0	NA	NA	351 (92.86)	NA	NA	286 (94.39)
M1	NA	NA	27 (7.14)	NA	NA	17 (5.61)
Lauren Subtype						
intestinal	265 (47.15)	NA	NA	NA	NA	260 (85.81)
mixed	51 (9.07)	NA	NA	NA	NA	26 (8.58)
diffuse	246 (43.77)	NA	NA	NA	NA	11 (3.63)
Fustat						
dead	283 (50.36)	209 (48.27)	247 (65.34)	189 (63.42)	21 (77.78)	151 (49.83)
alive	289 (49.64)	224 (51.73)	131 (34.66)	109 (36.58)	6 (22.22)	152 (50.67)

NA, Lack of information; T, tumor size; N, lymph node metastasis; M, distant metastasis.

### Construction of the TIIC model

We combined three GEO datasets (GSE62254, GSE57303, and GSE15459) to form a 562-sample dataset. The “ComBat” algorithm in the sva package was used to correct for batch effects in the GEO datasets. Using the R package glmnet, we performed LASSO regression to identify the most useful prognostic markers among these immune cell signatures in the GEO 562 cohort. The analysis filtered out 12 immune cells with nonzero coefficients, and a 10-fold cross-validation determined the best lambda value. We next used stepwise regression analysis to eliminate immune cells that are not valuable and highly related to other variables and to reduce the degree of multicollinearity. Finally, five immune cells were included in the TIIC model. Ultimately, the hazard coefficient obtained by stepwise regression analysis was multiplied by the normalized enrichment score (NES) of 5 immune cell markers and added with constant values to construct the TIIC model score. We validated the prognostic stability of this model in GSE84437, GSE62254, TCGA, and our clinical cohort.

### Evaluation of immune cell infiltration, GO, and KEGG pathway analyses

We used single-sample gene set enrichment analysis (ssGSEA) by the R package GSVA to quantify the infiltration levels of 24 immune cell signatures for each GC sample with mRNA expression data (12). NES reflects the relative abundance of each immune cell. We used the limma package to screen out differentially expressed genes between the two TIIC groups. KEGG and Gene Ontology (GO) term enrichment analyses were performed by using the R package ClusterProfiler to identify the biological pathways that the differentially expressed genes may be involved in (13). The R package heatplot was used to visualize the results.

### Clinical samples collection

Three hundred and three gastric cancer specimens were prepared in the tissue microarray (TMA) format, which were obtained from the Department of Clinical Biobank of the Affiliated Hospital of Nantong University. We collected clinicopathological information from the patients’ medical records. We screened the clinicopathological information of the patients and excluded cases with incomplete prognostic status as well as survival information. The patients had not received radiotherapy, chemotherapy, or biological immunotherapy before surgery. This research protocol was approved by the Human Research Ethics Committee of the Affiliated Hospital of Nantong University (Jiangsu, China) (14).

### Immunohistochemistry (IHC)

The paraffin-embedded TMA sections were placed in sodium citrate buffer (0.01 M, pH 6.0) for antigen retrieval. The primary antibody was incubated overnight in the 4°C refrigerator. DAB staining was performed after 10 minutes of secondary antibody incubation. We used hematoxylin for nuclear staining and xylene for sealing. The Vectra 3.0 automated quantitative pathology imaging system was used to scan the slides and detect and measure the positive rate of biomarkers. We trained machine learning algorithms to segment the images into tissue and interstitial parts and to accurately identify and quantify the phenotypes of those cells in all high-power fields within the entire tissue section (15).

### Immunofluorescent staining of tissues

Multiple immunofluorescence staining with multiple combinations was performed on the TMA sections. We first explored the staining conditions of every single antibody and then performed the staining combination to obtain the optimal multiple immunolabeling scheme. The details of immunofluorescent staining of tissues can be found in previous reports (14). The following antibodies were used in our research: rabbit anti–CD3 (85061S, Cell Signaling Technology), rabbit anti-CD4 (ab133616, Abcam), mouse anti-FOXP3 (ab20034, Abcam), mouse anti-CD45RO (55618S, Cell Signaling Technology), mouse anti-CD45RA (5788-2P200827, NOVUS), mouse anti-PD-1 (ab52587, Abcam), and anti-mouse cytokeratin (orb69073, Biobyt). The secondary antibody was Opal™ polymer HRP Ms+Rb (ARH1001EA, Perkin Elmer). DAPI (F6057, Sigma) was used to stain nuclei and seal the slides. We used the Opal 7-Color Manual IHC Kit (PerkinElmer, NEL811001KT) to detect the antibodies. TMAs were whole-slide imaged using inForm^®^ Cell Analysis software.

### Statistical analysis

Kaplan–Meier survival curves were generated by the R package survminer, and differences in overall survival between the two TIIC groups were evaluated by the two-sided log-rank test. The GC patients in the public cohorts were stratified into high- and low-TIIC score groups by using the optimal cutoff value acquired by X-tile software (16). We formulated a nomogram to provide a visual risk prediction based on TIIC scores and clinicopathological characteristics. A calibration plot was generated to assess the calibration ability of the nomogram. R software (v.3.6.0), Sangerbox 3.0 (17), and GraphPad Prism (v.5.0) were used to visualize images. The statistical analyses in our research were performed using SPSS (v.17.0). A P value less than 0.05 was considered statistically significant.

## Results

### Construction of the TIIC model

**Figure 1:**
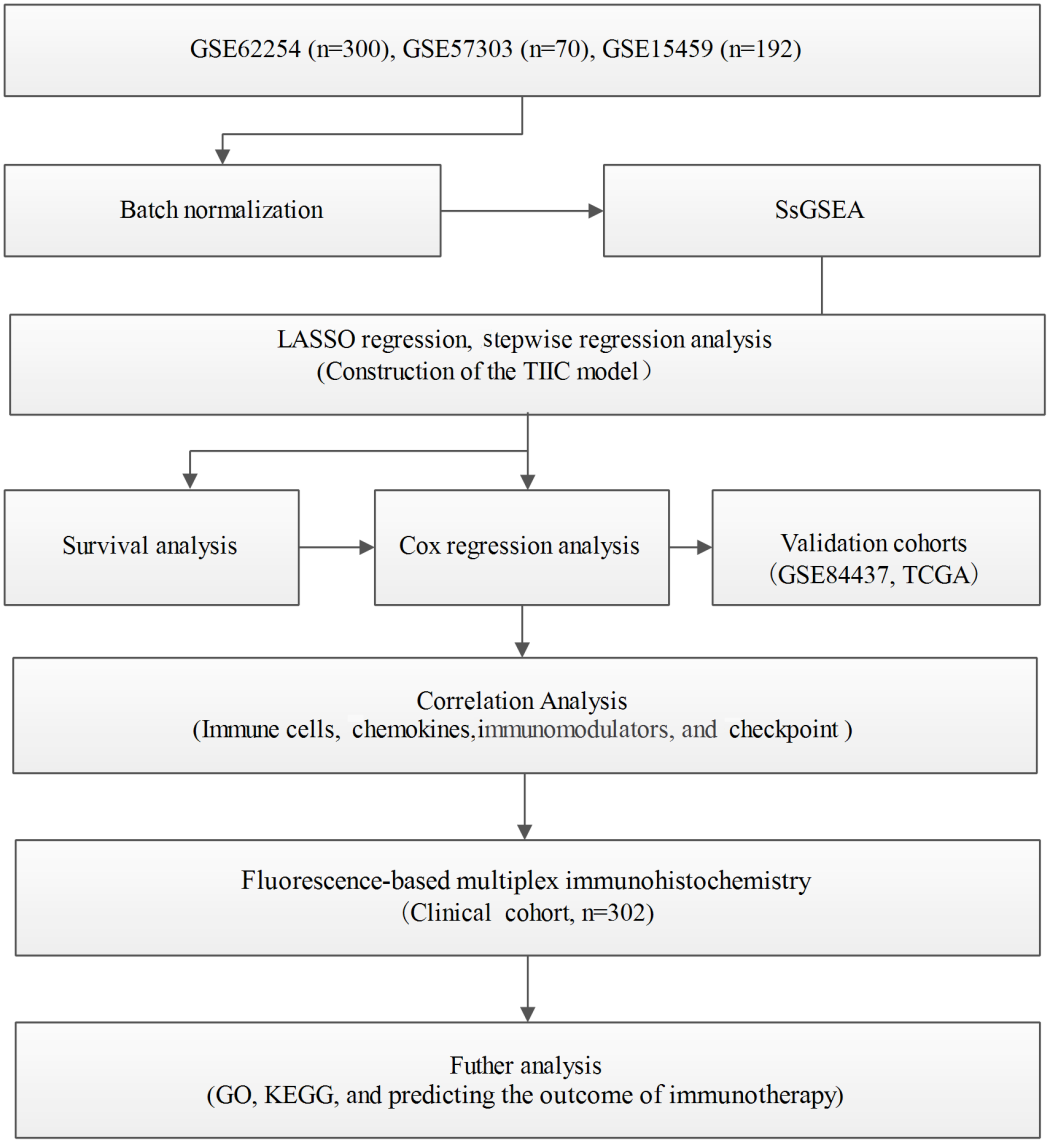
Workflow of the present study.

**Figure 2:**
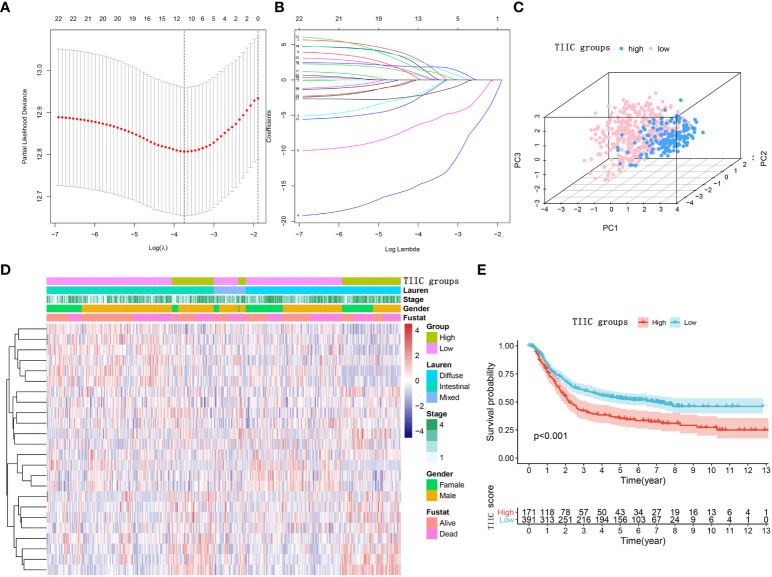
Construction of the TIIC model. **(A)** The LASSO regression model revealed partial likelihood deviance in the 10-fold cross-validation. The vertical dotted lines were drawn at the optimal values using the minimum and 1-SE criteria. **(B)** Twelve selected immune cell signatures in 10-fold cross-validation in the LASSO regression model. **(C)** Principal component analysis of the TIIC groups aimed to reveal the reliability of clustering. **(D)** Heatmap using the TIIC score from 24 immune cell signatures. The distribution of tumor-infiltrating immune cells in the TIIC groups. **(E)** Kaplan–Meier curve for the high- and low-TIIC score groups. Log-rank test.

Immunophenotyping can be applied to reflect the immune status of the tumor and its microenvironment, thus helping facilitate the development of subsequent immunotherapy (18). The comprehensive resource of genomic data in public databases offers a great opportunity to further understand the tumor immune microenvironment (19). The main flow of this research was presented in **Figure 1**. For exploring the underlying value of the immunophenotyping of gastric cancer (GC), 562 GC patients were used to quantify the enrichment levels of immune cells in GC tissues by the ssGSEA algorithm. Through the LASSO regression model, we selected 12 immune cell signatures (**Figures 2A, B**). We carried out stepwise regression analysis to eliminate immune cells that were not valuable and that were highly related to other variables and to reduce the degree of multicollinearity. Finally, five immune cells were included in this model. Our TIIC model had predictive strength, with a coefficient of determination of 0.646 and a P value of less than 0.001 (**Supplementary Table 1**). Based on the optimal cutoff point of the TIIC score, GC patients were stratified into low- and high-TIIC score groups to compare the differences in overall survival. The principal component analysis illustrated a significant distinction in the low- and high-TIIC score groups (**Figure 2C**). Utilization of unsupervised hierarchical cluster analysis revealed the distribution of tumor-infiltrating immune cells in the TIIC groups (**Figure 2D**). The TIIC model made up of five immune cells showed that the high-TIIC score group had an inferior prognosis compared with the low-TIIC score group (**Figure 2E**).

### The prognostic roles of the TIIC groups

**Figure 3:**
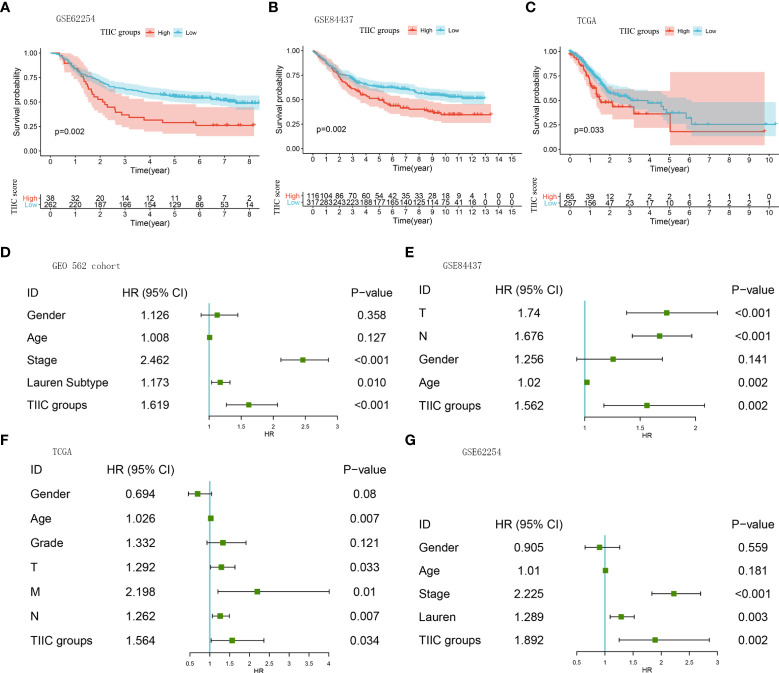
The prognostic roles of the TIIC groups. **(A–C)** Kaplan–Meier curves for the high- and low-TIIC score groups (GSE62254, GSE84437, TCGA-STAD). Log-rank test. **(D–G)** A forest plot visualized the impact of clinicopathological features and TIIC groups on overall survival, as evaluated using Cox univariate analysis (GEO 562 cohort, GSE84437, TCGA-STAD, and GSE62254).

Immune cells are well known to predict prognosis in several human cancers (20). Subsequently, we performed survival analysis and univariate Cox regression in multiple datasets to assess the stability of the TIIC groups in predicting the prognosis of GC patients. The high-TIIC score group had an inferior prognosis compared with the low-TIIC score group (GSE62254, GSE84437, and TCGA) (**Figures 3A–C**). Univariate Cox regression analysis included TIIC groups, sex, age, tumor stage, grade, and Lauren subtype, confirming that TIIC groups were an independent prognostic factor for GC patients (**Figures 3D–G**). The risk heatmap showed that survival deteriorated as the TIIC score increased (**Supplementary Figure 1A**). The immune cell infiltration degree differed between the high- and low-TIIC score groups. Compared with central memory cells, exhausted cells, nTregs, CD4+ T cells, and naïve CD4+ cells had a higher degree of infiltration in the high-TIIC score group. To provide a clinical diagnosis with a measurable method for predicting the overall survival of GC patients, we used the “rms” package to construct a nomogram that could predict 1-, 3- and 5-year overall survival by the TIIC score and other clinicopathologic features (**Supplementary Figure 1B**). The 1-, 3- and 5-year overall survival probability calibration curves revealed that the survival rate probability could be predicted well (**Supplementary Figures 1C–E**).

### Analysis of immune infiltration in the TIIC groups

**Figure 4:**
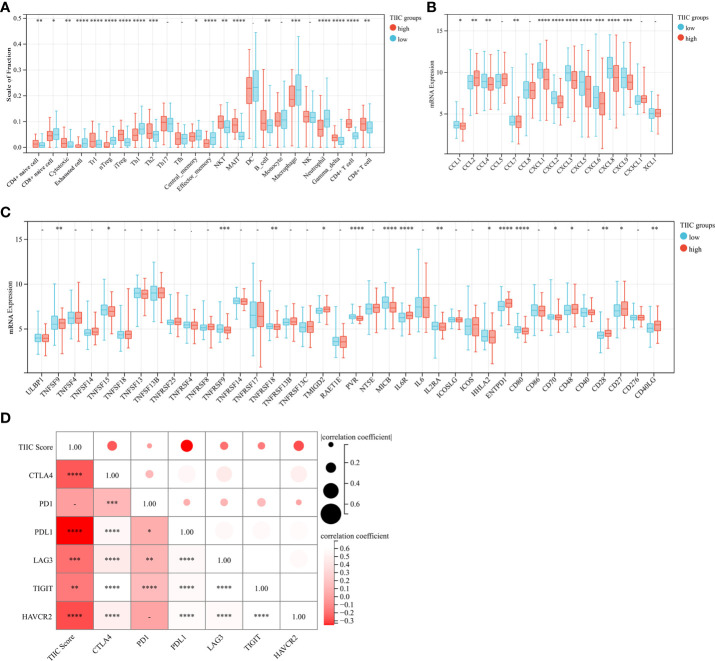
Immune cells, chemokines, immunomodulators, and checkpoints between high- and low-TIIC groups. **(A)** The fraction of immune cells in the TIIC groups. Within each group, the scattered dots represent immune cell expression values. Independent two-sample t-test. **(B)** Differential expression of common chemokines between the high- and low-TIIC score groups. Independent two-sample t-test. **(C)** The immunomodulators between high- and low-TIIC groups. Independent two-sample t-test. **(D)** The heatmap shows a relationship between each immune checkpoint, as well as between immune checkpoints and the TIIC score. Ns, P > 0.05; *P < 0.05; **P < 0.01; ***P < 0.001, ****P < 0.0001.

Aiming to explore and understand the biological behavior differences between the high- and low-TIIC score groups, we focused on the infiltration differences of 24 immune cells in the GEO 562 cohort (Composed of GSE62254, GSE57303, and GSE15459). The high-TIIC score group was characterized by the increased infiltration of CD4+ naïve cells, cytotoxic cells, Tr1 cells, iTregs, Th2 cells, NKT cells, MALT cells, B cells, gamma-delta cells, CD4+ T cells, and CD8+ T cells, whereas the low-TIIC score group was marked by the high infiltration of CD8+ naïve cells, exhausted cells, nTregs, Th1 cells, central memory cells, effector memory cells, macrophages, and neutrophils (**Figure 4A**). Cancer immune activity is a direct and integrated reflection of the function of the chemokine system and certain immunomodulators (21, 22). Several chemokines and immunomodulators were significantly different between the high- and low-TIIC score groups (**Figures 4B, C**). In addition, the heatmap demonstrated a close correlation between each immune checkpoint, as well as between immune checkpoints and the TIIC score (**Figure 4D**).

### The relationship between the TIIC scores, tumor subtypes, and somatic variations

**Figure 5:**
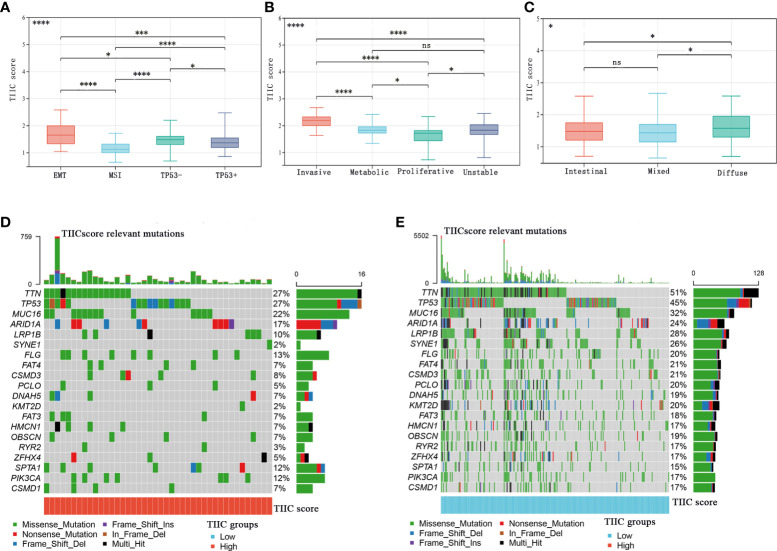
The relationship between the TIIC scores and tumor subtypes and somatic variations. **(A–C)** The distribution of TIIC scores in ACRG subtypes **(A)**, GSE15459 subtypes **(B)**, and Lauren subtypes **(C)**. Kruskal–Wallis test. **(D, E)** The InfoPrint was constructed using the high-TIIC score group on the left (red) and the low-TIIC score group on the right (blue). Ns, P > 0.05; *P < 0.05; **P < 0.01; ***P < 0.001, ****P < 0.0001.

The Asian Cancer Research Group (ACRG) confirmed the four molecular subtypes of gastric cancer and the survival time and recurrence rate of patients with different molecular subtypes were significantly different (23). The MSI subtype has a favorable prognosis, followed by the TP53+ and TP53- subtypes, and the EMT subtype has the worst prognosis (24). Consistent with previous discoveries, the TIIC score of the EMT subtype was significantly higher than that of other subtypes in the GSE62254 cohort (**Figure 5A**). In the GSE15459 cohort, tumors with high TIIC scores often tended to develop invasive types (**Figure 5B**). The Lauren classification is the most widely used in clinical practice and trials. According to histological characteristics, three types of gastric cancer are considered: intestinal type, diffuse type, and mixed type (25). Intestinal-type GC has higher rates of CD8+ cells, NK cells, and Tregs than diffuse/mixed-type GC (25). In our research, diffuse-type GC had higher TIIC scores than intestinal/mixed-type GC (**Figure 5C**). Furthermore, we evaluated the distribution of somatic variations in GC-driven genes between the high- and low-TIIC score groups in the TCGA cohort. The waterfall plot displayed the top 20 driver genes with the highest frequencies of alteration (**Figures 5D, E**).

### Multiplex fluorescence immunohistochemistry (mIHC)

**Figure 6:**
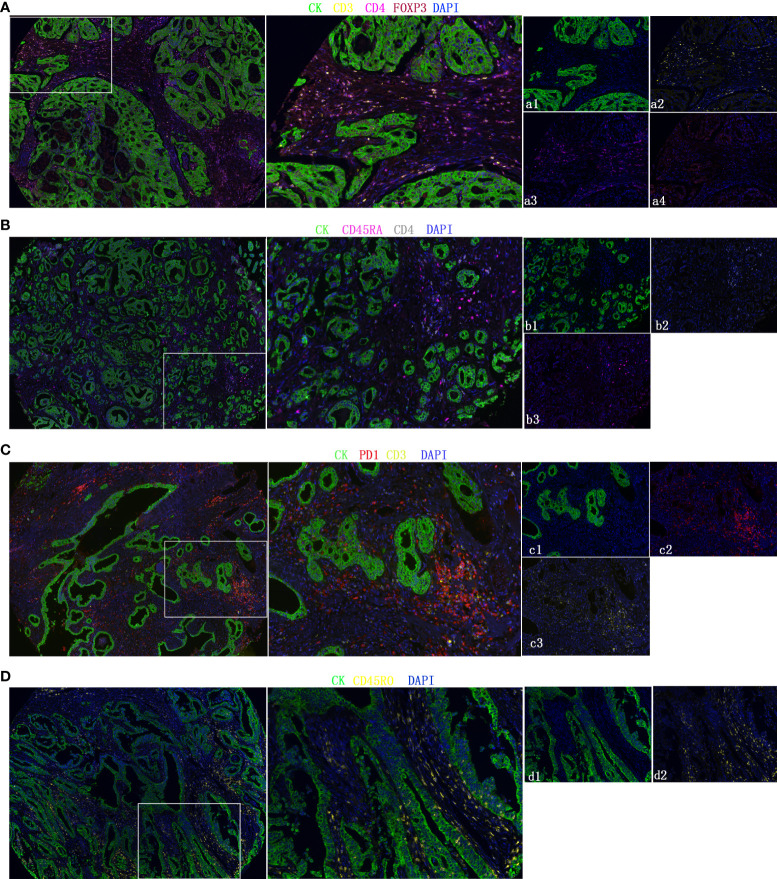
Representative fluorescence-based multiplex immunohistochemistry images. **(A–D)** CD4+ T cells (CD3, CD4), nTregs (CD4, FOXP3), CD4+ naïve cells (CD4, CD45RA), exhausted cells (CD3, PD-1), and central memory T cells (CD45RO). Cytokeratin (CK) was used to identify epithelial cells in tumor samples and define the tumor and stroma. All images were obtained using 20× zoom and were scaled digitally.

Only part of the transcriptomics data can be translated into the protein level, and the relative infiltration level of immune cells obtained based on the mRNA sequence does not represent the immune cells in the tumor well (26). We used clinical samples to explore the protein expression levels of these five immune cells. The mIHC method, which can accurately calculate immune cells in the microenvironment, was applied to the TMA of 302 GC patients. Representative immune markers were used to describe the infiltration levels of immune cells in the tumor microenvironment, for example, CD4+ T cells (CD3, CD4) (**Figure 6A**), nTregs (CD4, FOXP3) (**Figure 6A**), CD4+ naïve cells (CD4, CD45RA) (**Figure 6B**), exhausted cells (CD3, PD-1) (**Figure 6C**), and central memory T cells (CD45RO) (**Figure 6D**). The TIIC model composed of five immune cells showed that the high-TIIC score group also had an inferior prognosis compared with the low-TIIC score group in our research (**Supplementary Figure 2A**). Compared with other clinicopathological parameters, including Lauren subtype, gender, age, tumor size (T), lymph node metastasis (N), distant metastasis (M), TNM stage, tumor differentiation, preoperative serum carcinoembryonic antigen (CEA) levels, and preoperative serum carbohydrate antigen 19-9 (CA19-9) levels, univariate Cox regression revealed that the TIIC groups were indeed prognostic factors for gastric cancer (**Supplementary Figure 2B**). We also have done ROC curve analysis in our own queue and in the TCGA queue (**Supplementary Figures 2C, D**).

### The role of the TIIC model in the prediction of immunotherapeutic benefits

**Figure 7:**
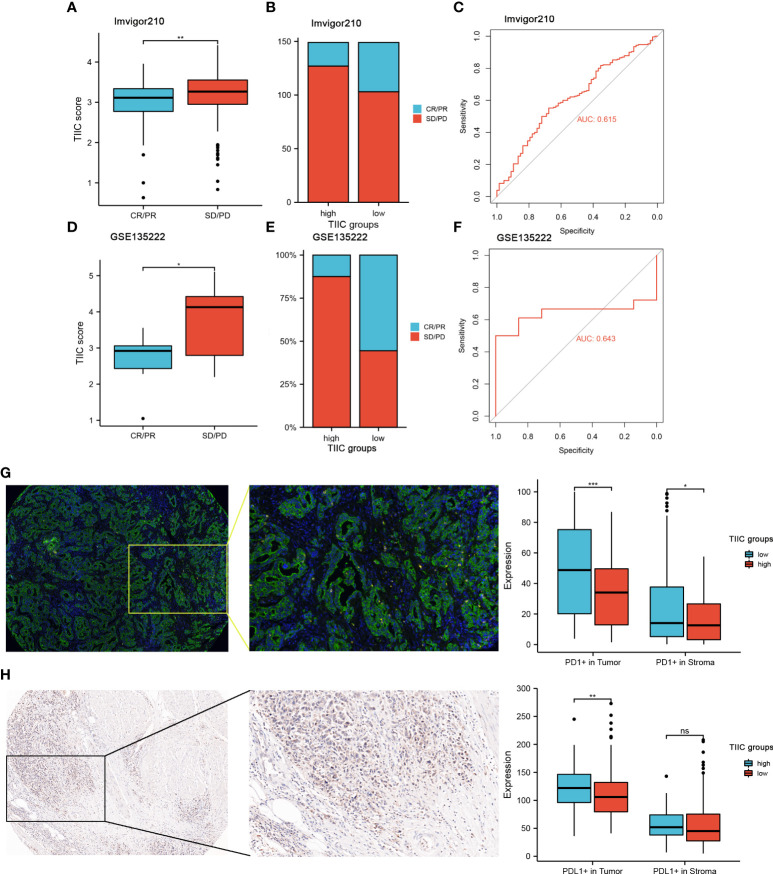
The value of the TIIC model in the prediction of immunotherapeutic benefits. **(A)** TIIC scores in groups with different anti-PD-1 clinical response statuses. Kruskal–Wallis test. **(B)** Rate of clinical response (complete response [CR]/partial response [PR] and stable disease [SD]/progressive disease [PD]) to anti-PD-L1 immunotherapy in the high- and low-TIIC score groups in the IMvigor210 cohort. **(C)** ROC curve measuring the sensitivity of the TIIC score in predicting the survival status of patients in the IMvigor210 dataset. **(D)** Distribution of TIIC scores in different response statuses to immunotherapy in the GSE135222 cohort. Kruskal–Wallis test. **(E)** Rate of clinical response (CR/PR and SD/PD) to anti-PD-1 immunotherapy in the high- and low-TIIC score groups in the GSE135222 cohort. **(F)** ROC curve measuring the sensitivity of the TIIC score in predicting the survival status of patients in the GSE135222 cohort. **(G-H)** mIHC and IHC revealed differences in PD-1 and PD-L1 expression between the high- and low-TIIC score groups. Ns, P > 0.05; *P < 0.05; **P < 0.01; ***P < 0.001.

In the subsequent analysis, we wanted to explore whether the TIIC model has predictive value for the immunotherapy response of GC patients. Public databases lack GC patients receiving immunotherapy. We used a dataset of urothelial carcinoma treated with anti-PD-L1 (Imvigor210) (progressive disease (PD), stable disease (SD), partial response (PR), and complete response (CR)) and a dataset of non-small cell lung carcinoma patients treated with anti-PD-1/PD-L1 (GSE135222). Patients with low TIIC scores were more likely to benefit from immune checkpoint therapy (**Figures 7A, D**). Compared with the high-TIIC score group, there was a mild increase in patients with complete or partial responses in the low-TIIC score group (**Figures 7B, E**). The area under the curve (AUC) of the TIIC model for predicting responsiveness to immunotherapy was 0.615 (Imvigor210) (**Figure 7C**) and 0.637 (GSE84437) (**Figure 7F**). The abundance of PD-1 expression between individual tumor types and within the same tumor type was associated with anti-PD-1 efficacy (27). We examined the protein expression levels of tumor and stromal PD-1 and PD-L1 in clinical samples (**Figures 7G, H**). The expression levels of PD-1 and PD-L1 were higher in the low-TIIC score group than in the high-TIIC score group in both the tumor and the stroma, which may account for the responsiveness of the low-TIIC score group to immunotherapy.

We then conducted GO and KEGG pathway analyses, and the heatmap presented the top 30 significantly enriched pathways (**Supplementary Figures 3A, B**). The results suggested that the TIIC groups may be involved in several immune-related pathways, including immune response−activating signal transduction, immune system process, activation of immune response, regulation of immune system process, adaptive immune response, Th17 cell differentiation, and Th1 and Th2 cell differentiation.

## Discussion

Currently, the immune microenvironment has been quantitatively analyzed. The tumor-infiltrating lymphocytes and composition of tumor-infiltrating lymphocytes are correlated with the patient outcome and the therapeutic response to immunotherapy (28). For example, CD4+ T cells and CD8+ T cells are associated with increased survival and response to immunotherapy. Tumor-infiltrating immune cells are vital in tumorigenesis, with a two-sided influence that regulates the immunosurveillance of cancer and creates a favorable microenvironment for cancer cell survival (29). Therefore, the quantitative evaluation of tumor-infiltrating lymphocytes may provide a new method for better stratification and for predicting the prognosis of gastric cancer (30).

Our research used the ssGSEA algorithm to evaluate the relative quantitative infiltrating levels of immune cells in a meta-cohort of 562 GC samples. We determined a robust prognostic tumor-infiltrating immune cell (TIIC) model. The cutoff based on the TIIC score can significantly stratify the overall survival rate of GC patients from the public cohort. Meanwhile, the high TIIC group was associated with worse survival in GC patients. We successfully confirmed the predictive ability of the TIIC model in multiple public cohorts. Furthermore, we performed fluorescence-based multiplex immunohistochemistry to confirm the prognostic value of the TIIC model in a series of tissue microarrays. Compared to clinical characteristics, we confirmed that the TIIC subtype was an independent prognostic factor for GC patients.

Moreover, the TIIC scores were also closely related to tumor subtypes and somatic variations. Due to the epidemiology, tumorigenesis, pathology, and molecular characteristics of cancer, gastric cancer, as a heterogeneous cancer, has multiple subtypes (25, 31). The research results of tumor classification are conducive to the development of clinical tumor treatment drugs. Our findings reveal the distinction in immune cell composition of tumor subtypes. We used 308 clinical samples to explore the protein expression levels of the five immune cells in the TIIC model. Interestingly, following the predicted prognosis of the public cohort, we confirmed the predictive value of the TIIC score in our clinical cohort.

The recruitment of naïve CD4+ T cells can lead to the immunosuppression of breast cancer (32). Studies have shown that the abundance of naïve CD4+ T cells is closely related to Tregs, which indicates that breast cancer patients have a poor prognosis (32). Some tumors with high expression of chemokine receptors can attract infiltration of suppressive immune cells, thus favoring their growth (6). In our study, chemokines were significantly different between the high- and low-TIIC score groups. We also found a significant correlation between the TIIC scores and the expression of several common immune checkpoints. The expression of immune checkpoints will cause the depletion of immune cells and immune escape of tumor cells and promote tumor progression (33). At present, various immune checkpoint inhibitors also mainly act on T cells to exert antitumor functions, so it is of utmost importance to reveal the functions of T cells infiltrated by tumors. CD4- naïve T cells and CD4+ T cells are enriched in high-risk subtypes, indicating an immunosuppressive tumor microenvironment. Therapeutic antibodies that block immune checkpoint pathways can induce strong and durable responses in various cancer patients (31, 34, 35). Immune checkpoint inhibitors have achieved good responses in many tumors. Tumor-infiltrating immune cells play a crucial role in checkpoint inhibitor immunotherapy (36). Some researchers roughly divide the tumor into “cold” tumors and “hot” tumors according to the characteristics of immune cell infiltration (37). Due to the diverse number and proportion of immune cells infiltrating the tumor, the response to immunotherapy also varies. Here, we utilized the IMvigor210 cohort to evaluate patients receiving immunotherapy and found that the TIIC score was significantly elevated in patients who did not respond to immunotherapy, which confirmed its predictive value. We also validated the expression levels of PD-1 and PD-L1 in our clinical samples. Overall, our research suggests that PD-1/PD-L1 dual blockade might be more suitable for patients with low TIIC scores.

Our research had some limitations. There may be remarkable heterogeneity among GC patients from different public datasets used in our study. The TIIC score only used a series of immune gene characteristics, and these characteristics cannot sufficiently represent the immune cells of the immune microenvironment. We did not explore the specific interaction mechanism of immune cells in the immune microenvironment. In subsequent work, we will collect information from GC patients who have undergone immunotherapy to further explore the influence of the TIIC score on therapeutic benefits. Many solid tumors are associated with immune cell infiltration in the tumor immune microenvironment, which affects therapy efficacy and overall survival to no small extent, yet the predictive role of individual cells on tumor prognosis is limited (38, 39). It is hypothesized that the integration of multiple immune cells may improve the prediction of prognosis for GC patients. However, current techniques to detect the level of immune cell infiltration in the tumor microenvironment of patients remain difficult. The clinical translation of this study is challenging and deserves future reflection and exploration.

In conclusion, the TIIC score can be clinically meaningful for predicting prognosis, thereby aiding in the assessment of the response of GC patients to PD-1/PD-L1 immunotherapy.

## Authors contributions

Conceptualization, JH; Data acquisition, SJ; Formal analysis, XD and QW; Funding acquisition, JH; Methodology, TC and MX; Visualization, SJ; Writing – original draft, SJ; Writing – review & editing, SJ. All authors contributed to the article and approved the submitted version.

## Data availability statement

The original contributions presented in the study are included in the article/**Supplementary Material**. Further inquiries can be directed to the corresponding author.

## Ethics statement

The study involving human subjects was reviewed and approved by the Human Research Ethics Committee of the Affiliated Hospital of Nantong University. Written informed consent to participate in this study was not required from the subjects in accordance with the national legislation and the institutional requirements.

## Funding

This work was supported by the National Natural Science Foundation of China (81874067) and the Technological Innovation and Demonstration of Social Undertakings Project fund (2022) of Nantong, Jiangsu, China.

## Acknowledgments

We sincerely appreciate all lab members.

## Conflict of interest

The authors declare that the research was conducted in the absence of any commercial or financial relationships that could be construed as a potential conflict of interest.

## Publisher’s note

All claims expressed in this article are solely those of the authors and do not necessarily represent those of their affiliated organizations, or those of the publisher, the editors and the reviewers. Any product that may be evaluated in this article, or claim that may be made by its manufacturer, is not guaranteed or endorsed by the publisher.
