# Supplementary material for: Identifying immune cells-related phenotype to predict immunotherapy and clinical outcome in gastric cancer
Source: Front Immunol. 2022 Aug 11;13:980986. doi: 10.3389/fimmu.2022.980986 (PMC9402937; doi:10.3389/fimmu.2022.980986)
Supplement: Supplementary file 1 [file Table_1.docx]

Supplementary Table1 Stepwise regression analysis

| Model | Unstandardized Coefficients | | Standardized Coefficients | | | t | P | Adjusted R Square | F |
| --- | --- | --- | --- | --- | --- | --- | --- | --- | --- |
|  | ꞵ | Std.Error | | ꞵ | |  |  |  |  |
| Constant | 0.795 | 0.046 | | - | 17.286 | | <0.001 | 0.642 | F (5,556) = 202.643,  P<0.001 |
| CD4_naive | 2.042 | 1.001 | | 0.054 | 2.041 | | 0.042 |  |  |
| Exhausted | -1.75 | 0.695 | | -0.069 | -2.519 | | 0.012 |  |  |
| nTreg | 2.587 | 0.674 | | 0.108 | 3.838 | | <0.001 |  |  |
| Central_memory | -2.359 | 0.54 | | -0.117 | -4.37 | | <0.001 |  |  |
| CD4_T | 13.545 | 0.511 | | 0.779 | 26.482 | | <0.001 |  |  |

R: the coefficient of determination, t: Statistics for the F-test, F: Statistics for the F-test.
