# Supplementary material for: Identifying immune cells-related phenotype to predict immunotherapy and clinical outcome in gastric cancer
Source: Front Immunol. 2022 Aug 11;13:980986. doi: 10.3389/fimmu.2022.980986 (PMC9402937; doi:10.3389/fimmu.2022.980986)
Supplement: Supplementary file 2 [file DataSheet_1.docx]

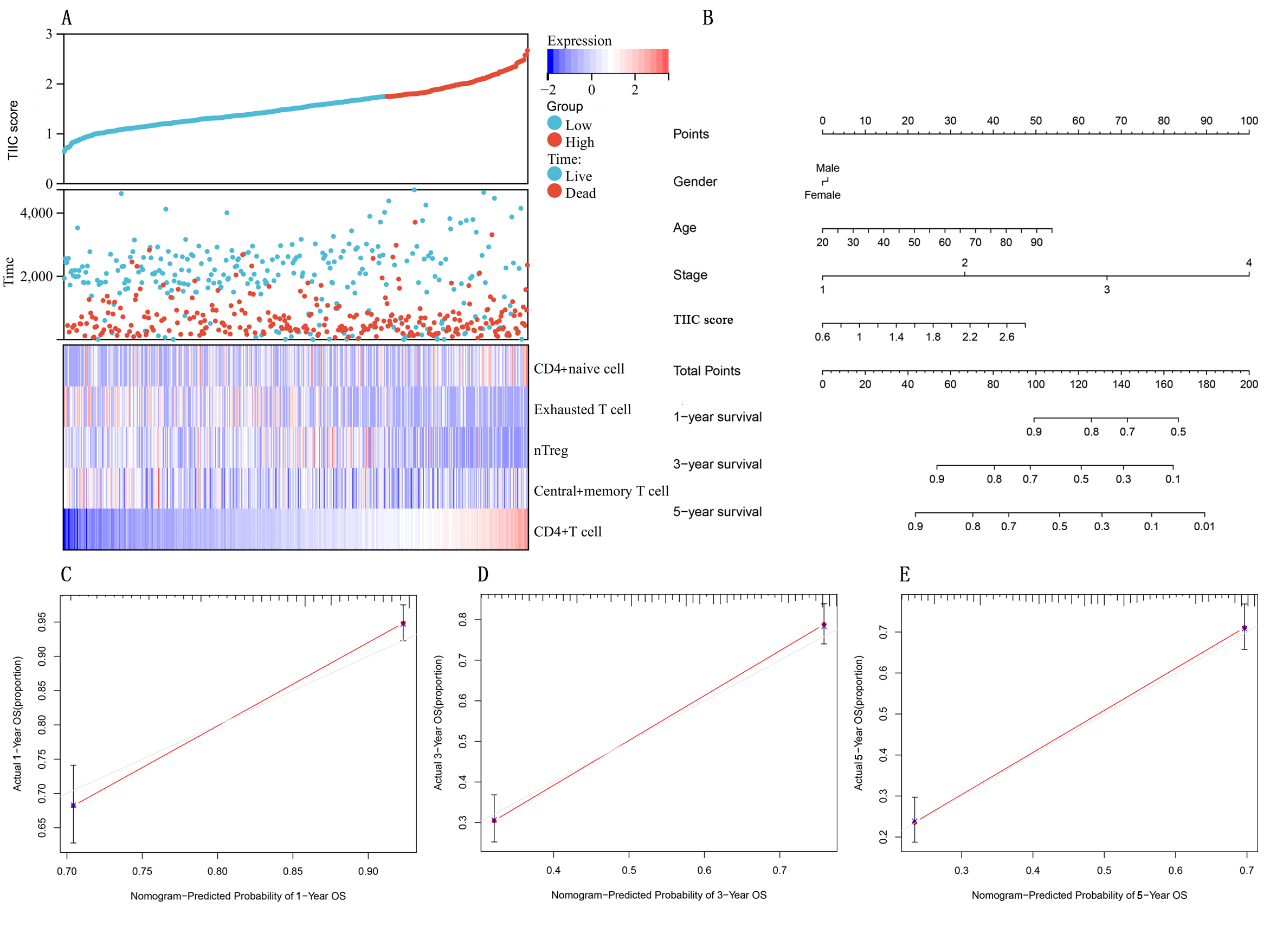


**Supplementary Figure S1** **(A)** TIIC score curve showing the distribution of the TIIC score in the GEO 562 cohort. Distribution of survival statuses and TIIC scores in the GEO 562 cohort. Heatmap of the five immune cells for GC patients with high and low TIIC scores. **(B)** The nomogram was constructed with TIIC scores and incorporated clinicopathologic features. **(C-E)** Calibration plot of the nomogram in terms of the agreement between the predicted and observed 1-, 3- and 5-year outcomes.


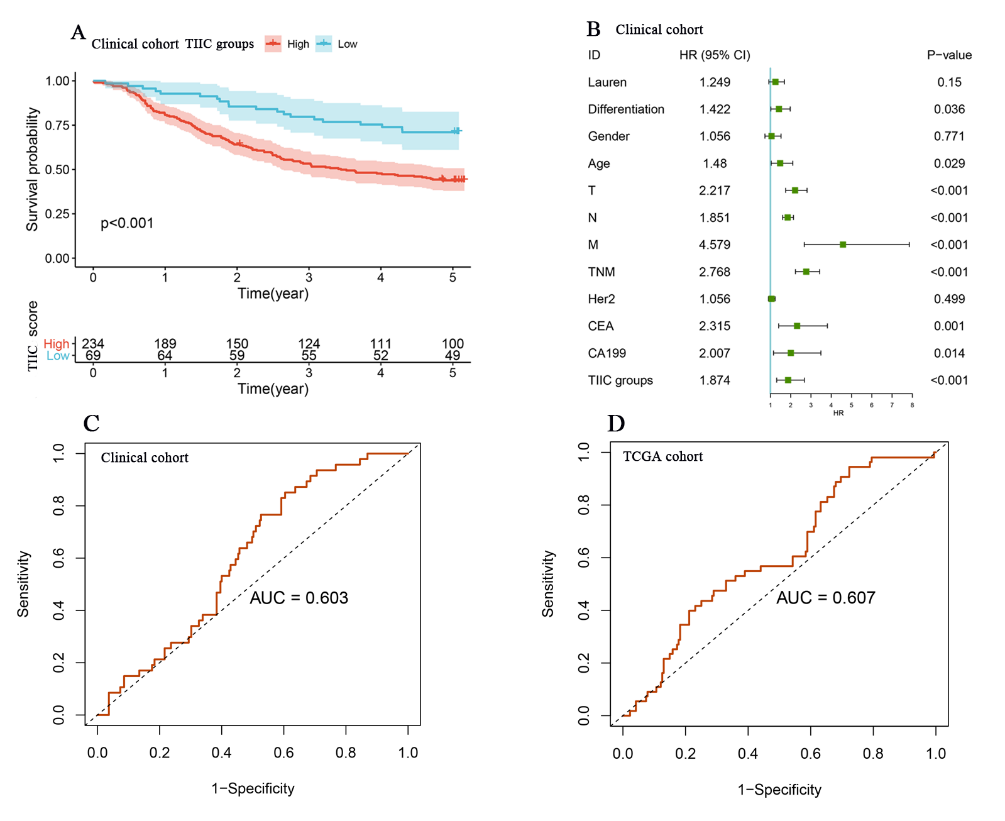


**Supplementary Figure S2 (A****)** Kaplan–Meier curves for the high and low TIIC score groups in our clinical cohort. (**B)** A forest visualized the impact of clinicopathological features and TIIC groups on overall survival, as evaluated using Cox univariate tests in our clinical cohort. **(C-D)** ROC curve analysis of prognosis prediction by the TIIC scores.


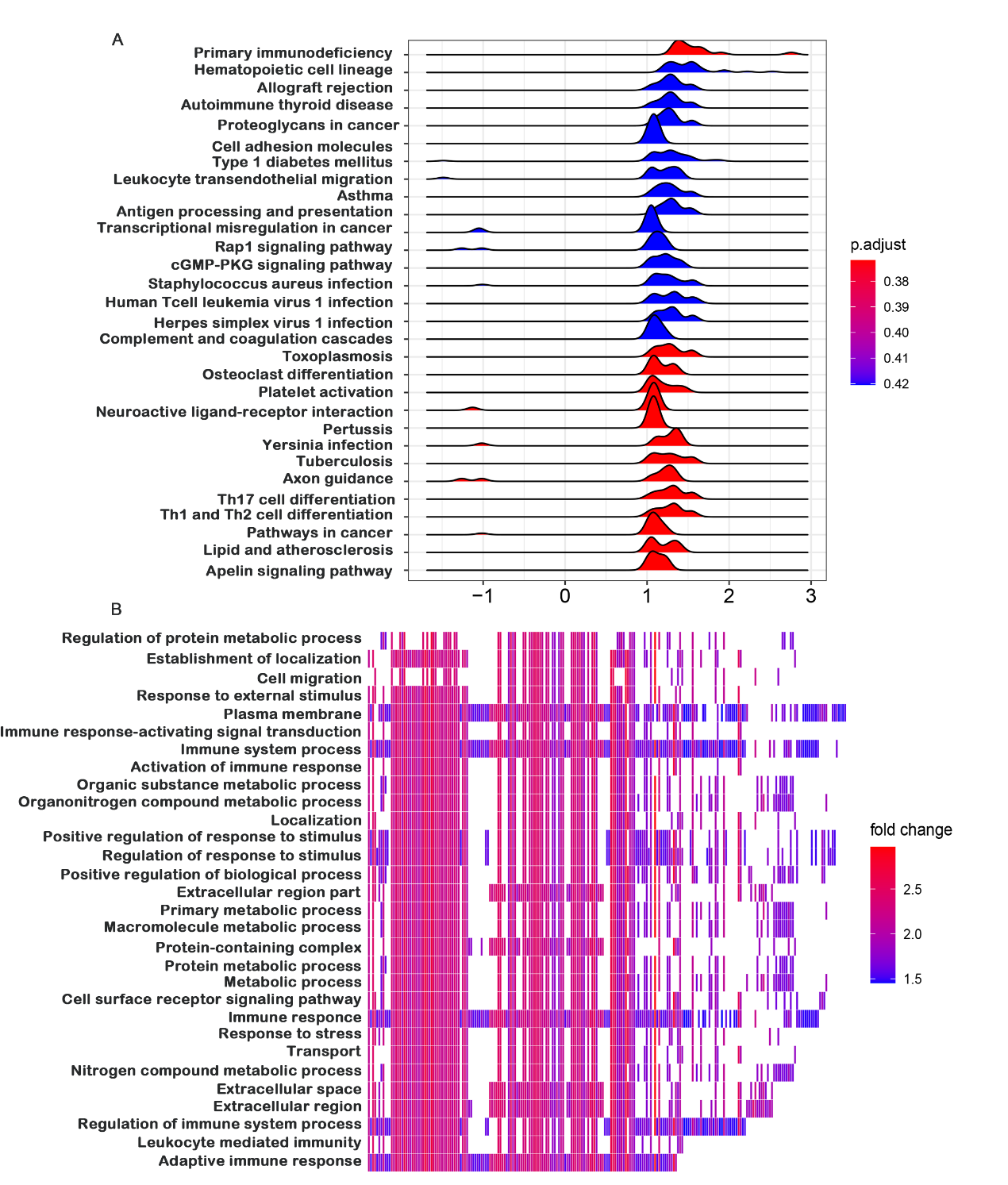


**Supplementary Figure S3. (A-B)** Visualization of the top 30 enriched biological processes and the top 30 enriched KEGG pathways by the DEGs in the low versus high TIIC scores groups.
